# Met1-linked ubiquitination in immune signalling

**DOI:** 10.1111/febs.12944

**Published:** 2014-08-12

**Authors:** Berthe K Fiil, Mads Gyrd-Hansen

**Affiliations:** 1 Department of Disease Biology, Novo Nordisk Foundation Centre for Protein Research, Faculty of Health and Medical Sciences, University of CopenhagenDenmark; 2 Ludwig Institute for Cancer Research, Nuffield Department of Clinical Medicine, University of OxfordUK

**Keywords:** immune receptor signalling, inflammation, innate immunity, LUBAC, Met1-linked ubiquitin, NEMO, OTULIN

## Abstract

N-terminal methionine-linked ubiquitin (Met1-Ub), or linear ubiquitin, has emerged as a central post-translational modification in innate immune signalling. The molecular machinery that assembles, senses and, more recently, disassembles Met1-Ub has been identified, and technical advances have enabled the identification of physiological substrates for Met1-Ub in response to activation of innate immune receptors. These discoveries have significantly advanced our understanding of how nondegradative ubiquitin modifications control proinflammatory responses mediated by nuclear factor-κB and mitogen-activated protein kinases. In this review, we discuss the current data on Met1-Ub function and regulation, and point to some of the questions that still remain unanswered.

## Introduction

Infection and colonization by pathogens is a constant threat to humans and other multicellular organisms, and an effective immunological barrier between the inside of the organism and the surrounding environment is critical for human health. The innate immune system is the first line of defence against invading pathogens, and its activation relies on pattern recognition receptors (PRRs) that recognise molecular patterns on/in the microorganism, and on cytokine receptors that amplify the inflammatory response 1,2. PRRs and cytokine receptors (hereforth collectively referred to as innate immune receptors) are present on the cell surface and in the cytoplasm of immune cells and nonimmune cells 2,3. Stimulation of these receptors triggers kinase signalling pathways that lead to an inflammatory response mediated by nuclear factor-κB (NF-κB) transcription factors and transcription factors activated by mitogen-activated protein kinases (MAPKs) 1,4. These transcription factors regulate fundamental aspects of the immune response by driving the expression of cytokines, chemokines, antimicrobial effectors and genes that control cellular survival, proliferation, and migration/invasion 5.

The signalling processes triggered by innate immune receptors are controlled by conjugation of ubiquitin (Ub) chains onto components of the receptor signalling complexes 1. This is carried out by ubiquitin ligases (E3) that, together with E1 and E2 enzymes, assemble Ub chains internally linked via any of seven Lys residues or the N-terminal methionine (Met1) 6. This allows for the generation of at least eight types of Ub chain that adopt different topologies and constitute distinct signals within the cell 6.

In this review, we discuss the role and regulation of Ub chains assembled through Met1 [N-terminal methionine-linked Ub (Met1-Ub)], a Ub chain type whose cellular function(s) has come under intense investigation along with the discovery of cellular proteins dedicated to the assembly (termed ‘writers’), sensing (termed ‘readers’) and disassembly (termed ‘erasers’) of the Met1-Ub linkage. We focus on its role in innate immune receptor signalling and inflammatory processes, but it is worth noting that Met1-Ub has also been linked to other cellular processes, including survival signalling after DNA damage and restriction of Wnt signalling during embryonic development 7–9.

## Met1-Ub machinery

### Writers

**Fig 1:**
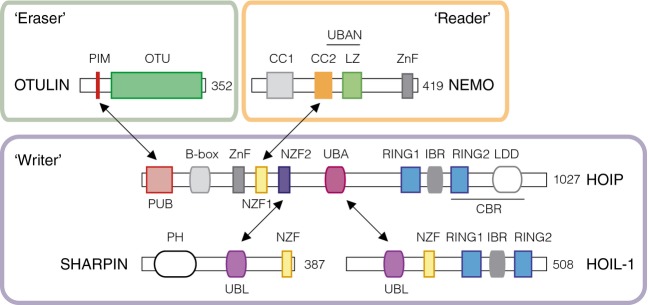
The Met1-Ub machinery. Schematic model of the proteins and their domain organisation involved in Met1-Ub assembly (Writer), Met1-Ub sensing (Reader), and Met1-Ub disassembly (Eraser). Double arrows indicate interacting domains. LZ, leucine zipper; PH, pleckstrin homology; ZnF, zinc finger.

Met1-Ub is produced by the genes *UBB* and *UBC*; in humans, these encode three and nine head-to-tail linked copies of Ub, respectively 10–12. The Met1-Ub molecules are processed cotranslationally by specialised deubiquitinases (DUBs) 13, providing free monomeric Ub for conjugation onto target proteins and for assembly into Lys-linked and Met1-linked Ub chains. The enzymatic assembly of Met1-Ub was first described by Iwai *et al*. in 2006, in a report identifying an approximately 600-kDa E3 complex containing the RING-in-between-RING proteins haem-oxidized IRP2 ubiquitin ligase-1 (HOIL-1; also termed RanBP-type and C3HC4-type zinc finger-containing 1 and RNF54) and HOIL-1-interacting protein (HOIP; also termed RNF31 and ZIBRA). The HOIP–HOIL-1 complex exclusively assembles Ub chains linked via Met1, and is termed the linear Ub chain assembly complex (LUBAC). Subsequently, SHANK-associated RH domain interactor (SHARPIN) was identified as a third core component of the LUBAC complex 14–16. HOIP is the catalytic subunit of the LUBAC complex, but is autoinhibited and requires binding of the HOIL-1 UBL domain to its Ub-associated (UBA) domain or the SHARPIN Ub-like (UBL) domain to its Npl4 zinc finger (NZF)2 domain for activity 14,17,18 (Fig. 1).

**Fig 2:**
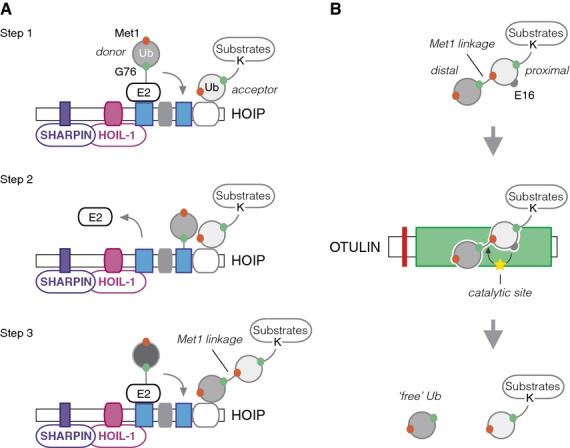
Assembly and disassembly of Met1-Ub. (A) Schematic model of Met1-Ub assembly by HOIP and the cofactors HOIL-1 and SHARPIN. HOIP is catalytically active when bound by HOIL-1 and/or SHARPIN. Cognate Ub-charged E2s (e.g. UbcH7) dock to RING1, which facilitates transfer of the Ub to RING2/CBR. The HOIP RING2-LDD/CBR orients the acceptor Ub, here shown conjugated to a Ub substrate (Step 1). The charged Ub is transferred from the E2 to Cys885 in RING2/CBR. The orientation of the acceptor Ub relative to the catalytic Cys in the CBR juxtaposes Met1 in the acceptor to the thioester bond between the donor Ub (G76) and Cys885 (Step 2). The donor Ub is subsequently conjugated to the α-amino group on Met1 of the acceptor to form a Met1 0linkage. This process can be repeated with a new Ub-charged E2 to extend the Met1-Ub chain further (Step 3). (B) Schematic model of how Met1 linkages are disassembled by OTULIN. Met1-Ub (here attached to a substrate) is bound by OTULIN's OTU. Glu16 of the proximal Ub inserts into the catalytic site of the OTU domain, which reorients the catalytic triad to enhance the hydrolytic activity of the enzyme, facilitating disassembly of the Met1 linkage. The Met1-Ub chains can be trimmed down to the Ub conjugated directly to a Lys in the substrate. This linkage is different from any Ub–Ub linkage, and is most likely less prone to hydrolysis by linkage-selective DUBs.

The region determining the chain-linkage specificity of HOIP is embedded in the C-terminal part of the protein, where the RING1 domain interacts with Ub-charged E2s to transfer Ub to Cys885 in the RING2 domain 17,19,20. Transfer of the donor Ub to Met1 of the acceptor Ub is facilitated by the RING2 domain and linear ubiquitin chain-determining domain (LDD) C-terminal to the RING2 domain 20 (Fig. 2). Structural studies indicate that the RING2 domain and LDD form part of one structural unit distinct from the cross-brace structure of RINGs, and this unit is therefore also referred to as catalytic in-between-RING (IBR) (CBR). CBR harbours the Cys residue that receives Ub from the E2, and in addition docks and positions the acceptor Ub to enable conjugation of the C-terminal Gly76 in the donor Ub to the α-amino group of Met1 on the acceptor Ub 19. These studies explain the molecular and structural underpinnings of how the LUBAC complex assembles Met1-Ub and no other Ub linkage.

### Erasers

Similarly to the genetically encoded Met1-Ub, LUBAC-assembled Met1-Ub is disassembled by dedicated DUBs. The recently discovered Met1-Ub ‘eraser’ ovarian tumour (OTU) domain DUB OTULIN (also termed Fam105B and Gumby) exclusively cleaves Met1-linked Ub chains 7,21. This specificity is achieved through its high affinity for the Met1 linkage, and through substrate-assisted formation of the catalytic site involving Glu16 in the proximal Ub 21 (Fig. 2). The proteolytic activity of OTULIN appears to be independent of the Ub-modified substrate, suggesting that it can also cleave genetically encoded Met1-Ub. In fact, mouse embryos carrying a mutation in OTULIN (OTULIN^W96R^) that predictably impairs its ability to interact with Met1-Ub 21 have increased steady-state levels of Met1-Ub 7, which implies a role for OTULIN in the general disassembly of Met1-linked Ub chains.

OTULIN interacts directly with a peptide: *N*-glycanase/UBA-containing or UBX-containing protein (PUB) domain in HOIP via an evolutionarily conserved PUB-interacting motif (PIM) in the N-terminal region preceding the OTU domain 22–24 (Fig. 1). Intriguingly, gel filtration experiments have suggested that a significant proportion of OTULIN associates with the LUBAC complex, but that this association can be abolished by phosphorylation of an invariant Tyr in the OTULIN PIM (Tyr56) 22,23. Although the kinases or phosphatases regulating the phosphorylation are unknown, this suggests an elegant mechanism for regulating LUBAC's capacity to assemble Met1-Ub.

The cylindromatosis tumour suppressor (CYLD) also disassembles Met1-linked Ub chains *in vitro* (in addition to Lys63-linked Ub chains), and has been shown to associate with the HOIP PUB domain 24. Taken together, these findings show that cellular Met1-Ub assembly is balanced by the opposing activities of LUBAC and OTULIN and possibly also CYLD (and other DUBs).

### Readers

Met1-Ub and Lys63-Ub adopt similar open conformations, unlike other linked chains, such as Lys48-linked and Lys11-linked chains, which adopt a compact closed topology 25–28. Nonetheless, Ub-binding domain-containing proteins that ‘read’ the Ub chains can, in several cases, discriminate between Met1-Ub and Lys63-Ub with remarkably high specificity. The best-described example is NF-κB essential modifier (NEMO; also termed inhibitor of NF-κB kinase), which binds Met1-Ub with high specificity, although it can also bind with low affinity to Lys63-Ub and to other Lys-linked chains 29–32. Specificity for the Met1 linkage is achieved through its Ub-binding in A20 binding and inhibitor of NF-κB (ABIN) and NEMO (UBAN) domain positioned within the coiled-coil (CC) and zinc finger (CoZi) region, which interacts with the canonical hydrophobic Ile44 patch and residues of the C-terminal region in the distal Ub, and residues in the Phe4 patch in the proximal Ub 29,31. Missense mutations in the UBAN domain affecting the binding to Met1-Ub (as well as other linkages) cause a severe disorder termed X-linked ectodermal dysplasia with immunodeficiency, characterised by defects in the development of teeth, hair, skin, and sweat glands, and susceptibility to pyogenic bacterial infections 29,33–35 (Table 1).

**Table 1:** Overview of the implications of genetic alterations in Met1-Ub machinery. fs, frameshift; X, nonsense mutation.

Genetic defect	Molecular defect	Phenotype/pathology	References
Human			
HOIP^Q584H^ HOIP^Q622L^	Stronger binding to HOIL-1 resulting in increased LUBAC activity	SNPs are enriched in activated B-cell-like DLBCL Increased BCR-mediated NF-κB activity	45
HOIL-1^L41fsX7^ HOIL-1^Q185X^	No detectable HOIL-1 Decreased levels of HOIP and SHARPIN	Susceptibility to pyogenic bacterial infections Systemic autoimmunity Muscular amylopectinosis	55
NEMO^D311N^ NEMO^E315A^ NEMO^R319Q^^a^	Loss of binding to Met1-Ub and other linkages	Ectodermal dysplasia Susceptibility to pyogenic bacterial infections	29,33–35
Mouse			
HOIP^C879S^	Loss of LUBAC catalytic activity	Lethality at embryonic day 11	44
HOIP^delRBR^ (in B cells)	Truncated and catalytically inactive HOIP Specific loss of LUBAC activity in B cells	Defect in development of B1 cells caused by defect in NF-κB and MAPK activation downstream of CD40 and transmembrane activator and CAML interactor	43
HOIL-1^−/−^	No detectable HOIL-1 Decreased levels of HOIP	No overt phenotype Slightly increased susceptibility to TNF-induced liver damage Protected from lipopolysaccharide-induced fatal peritonitis	41,56
*Cpdm* (frameshift mutations in *SHARPIN*)	No detectable SHARPIN Decreased levels of HOIP and SHARPIN	Chronic proliferative dermatitis mediated by TNF Multiorgan inflammation Defective development of secondary lymphoid organs	14,42
OTULIN^W96R^ OTULIN^D336E^	Loss of/reduced OTULIN activity Increased cellular level of Met1-Ub	Lethality at embryonic days 12.5–14 Defect in angiogenesis Improper branching of vascular networks in the head and trunk May be related to impaired Wnt signalling	7

a Ub binding defect has not been verified experimentally.

In ABIN proteins, the conserved UBAN domain also mediates binding to Met1-Ub, although ABINs have been shown to also bind Lys63-Ub 36–38. However, direct comparison of binding to Met1-Ub, Lys63-Ub and Lys48-Ub indicates that at least ABIN1 and ABIN2 strongly prefer Met1-Ub to the other linkages 26,29. Other proteins that have been found to interact with Met1-Ub include the inhibitor of apoptosis (IAP) proteins cIAP1/2 and X-linked IAP (XIAP), and the LUBAC subunits HOIL-1 and SHARPIN 15,26,39,40. However, as these proteins are also reported to bind other linkages, it is unknown how preferential is the binding to Met1-Ub.

## Immune signalling

### Tumour necrosis factor (TNF) receptor 1 (TNFR1)

**Fig 3:**
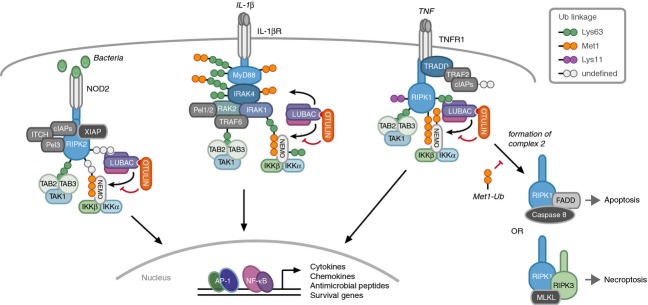
Model of innate immune signalling pathways controlled by Met-Ub. For NOD2 (left), RIPK2 is the central adaptor for assembly of the signalling complex and is the predominant target for ubiquitination. LUBAC is recruited through the Ub chains conjugated by XIAP, and LUBAC, in turn, conjugates Met1-Ub on existing Ub chains (or mono-Ub). The formation of Met1-Ub is essential for activation of NF-κB and MAPK pathways and for a productive transcriptional response. Conversely, OTULIN is needed to restrict accumulation of Met1-Ub that otherwise leads to an excessive transcriptional response. In IL-1β signalling (centre), MyD88 is the central adaptor for assembly of the signalling complex. In addition to MyD88, several other complex components are modified by Lys63-Ub. LUBAC extends the Lys63-Ub with Met1-Ub, thereby generating Lys63/Met1 mixed-linkage Ub chains. The specific Ub chains responsible for recruiting LUBAC have not been established. Notably, NEMO is predominantly modified by Lys63-Ub but binds Met1-Ub to enable productive signalling. In TNF signalling (right), cIAPs are responsible for recruiting LUBAC to the TNFR1 signalling complex. RIPK1 is a main target for ubiquitination, and is modified with Lys11-Ub, Lys63-Ub, and Met1-Ub. LUBAC conjugates Ub chains on RIPK1 and NEMO, and probably other complex components. This stabilises the signalling complex and enables optimal activation of NF-κB and MAPK pathways. In addition, Met1-Ub prevents the formation of cell death-inducing complexes that trigger either caspase-mediated apoptosis or RIPK3-mediated necroptosis. AP-1, activator protein-1; FADD, Fas-associated protein with Death Domain; ITCH, itchy E3 ubiquitin protein ligase; MLKL, Mixed Linkeage Kinase Domain-Like.

The TNFR1 signalling complex, also referred to as complex 1, was the first signalling complex found to contain LUBAC and to be regulated by its activity 39,41. LUBAC is recruited to TNFR1 complex 1 through Ub chains conjugated by cIAP1/2 on receptor-interacting protein kinase (RIPK)1 and possibly other proteins. In turn, LUBAC assembles Met1-Ub chains, which contribute to stabilisation of the signalling complex, allowing for full activation of MAPKs and the canonical IκB kinase complex (IKK), and transcription of NF-κB target genes 39,41 (Fig. 3). It is important to note that LUBAC is not a prerequisite for TNF-induced inflammatory signalling as such, but rather functions to enforce the process, which nonetheless is likely to be important for proper immune function. In agreement with this, the cellular level of OTULIN does not dramatically affect TNF-induced NF-κB activation or transcription of target genes 21,23.

Another role for LUBAC and Met1-Ub in TNF signalling is the regulation of cell death in response to TNFR1 stimulation. Ablation or depletion of HOIL-1 or SHARPIN favours apoptotic and necroptotic cell death after TNF treatment 14,15,39,41 (Table 1). Similarly, overexpression of OTULIN reduces the clonogenic survival of cells treated with TNF 21. The target for ubiquitination by LUBAC and the molecular function of Met1-Ub in preventing cell death signalling have not yet been determined. However, the importance of this role for Met1-Ub in the context of TNFR1 signalling is underscored by the fact that ablation of SHARPIN, which leads to diminished levels of HOIL-1 and HOIP in cells, causes chronic proliferative dermatitis in mice (termed the *cpdm* phenotype) 14,42 (Table 1). The mice show an increased level of cell death of keratinocytes and progressive inflammation, a phenotype that is reversed by ablation of TNF 14,15. Notably, ablation of TNF did not correct other immune disorders of the *cpdm* mice, which include multiorgan inflammation and defective secondary lymphoid organ development 42.

### CD40 and B-cell receptor (BCR)

LUBAC is also essential for a full inflammatory response from the CD40 receptor upon its activation by CD40 ligand or antibodies against CD40 14,16. CD40 is constitutively expressed by antigen-presenting cells, and a conditional knock-in mouse expressing a truncated (catalytically inactive) HOIP lacking the RING1–IBR–CBR region in B cells shows severely impaired B1-cell development 43 (Table 1). The inflammatory response to CD40 and transmembrane activator and CAML interactor (another TNF superfamily member) activation was impaired in B cells carrying the truncated HOIP, and this resulted in a severely impaired antibody response 43, perhaps explaining parts of the immunological phenotype of *cpdm* mice. It is of note that systemic knock-in of catalytically inactive HOIP is embryonically lethal 43,44 (Table 1).

BCR signalling was not impaired in murine B cells deficient in HOIP catalytic activity 43. In contrast to this, rare single-nucleotide polymorphisms (SNPs) in human *RNF31* (the gene encoding HOIP) that give rise to single amino acid substitutions in the HOIP UBA domain, increasing HOIL-1 binding and Ub ligase activity, were found to increase BCR signalling and NF-κB activity in activated B-cell-like subtype diffuse large B-cell lymphoma (DLBCL) cell lines 45. Conversely, depletion of HOIP in these cells decreased BCR signalling and reduced their viability, consistent with the dependence of DLBCL cells on constitutive NF-κB activity mediated by BCR and the essential B-cell and T-cell receptor signalling complex CARMA1–BCL10–MALT1 46,47. Intriguingly, the identified SNPs in *RNF31* were found to be enriched eight-fold in patients with DLBCL as compared with healthy individuals. This suggests that improper control of Met1-Ub assembly might be involved in the development of B-cell lymphoma 45 (Table 1).

### Interkeukin (IL)-1β receptor (IL-1βR)

Met1-Ub in IL-1βR signalling enables optimal and/or correct activation of kinase pathways and transcription of NF-κB target genes. Whereas the Ub linkage type(s) required for TNFR1 inflammatory signalling remain somewhat controversial, with no defined linkage type being essential, IL-1βR signalling relies largely on Lys63-Ub 48. The Ub ligase TNF receptor-associated factor (TRAF)6, which conjugates predominantly Lys63-Ub, is needed for IL-1β-induced activation of MAPKs and NF-κB, whereas the Lys63-specific E2 Ubc13 is needed only for MAPK activation 48–51. Interestingly, Emmerich *et al*. 44 demonstrated that Met1-Ub is formed on existing Lys63-linked Ub chains conjugated onto components of the IL-1βR signalling complex 44, presumably by the Ub ligases TRAF6 and/or Pellino 1/2 52,53 (Fig. 3). This observation might explain the strict dependency on Lys63-Ub in this system, as a loss of Lys63-Ub will also prevent the formation of Met1-Ub on certain targets.

It was recently reported that NEMO is recruited to higher-order structures visible by microscopy upon receptor activation 54. Consistent with the differential role for specific Ub linkages in TNF and IL-1β responses, the recruitment of NEMO to the structures after IL-1β treatment was dependent on HOIL-1 (as well as Lys63-Ub), whereas HOIL-1 was partially dispensable for the recruitment after TNF treatment.

The requirement for Met1-Ub in immune regulation is underscored by the identification of germline mutations in *RBCK1* (the gene encoding HOIL-1) in individuals suffering from susceptibility to pyogenic bacterial infections, systemic autoimmunity, and muscular amylopectinosis 55 (Table 1). Gene expression profiling in fibroblasts isolated from patients showed a blunted response to IL1β and, to a lesser extent, to TNF, which is in agreement with the described role for LUBAC and Met1-Ub in these signalling pathways. Surprisingly, gene expression profiling in monocytes from HOIL-1-deficient patients revealed a hyper-responsive phenotype as compared with monocytes from healthy donors 55. This appears to also be the case for mouse cells, as HOIL-1-deficient mouse embryonic fibroblasts have an impaired response to TNFR1 and Toll-like receptor (TLR)4 ligands, whereas HOIL-1-deficient bone marrow-derived macrophages respond similarly to wild-type cells 56. The underlying cause of the different responses of fibroblasts and monocytic cells is not known, but they suggest that Met1-Ub, like Lys63-Ub, serves not only receptor-specific functions but also cell type-specific functions in immune signalling.

### PRRs – nucleotide-binding and oligomerisation domain-containing protein (NOD)1 and NOD2

We and others recently demonstrated that LUBAC has a pronounced role in facilitating inflammatory signalling downstream of the PRRs NOD1 and NOD2 57,58. NOD1 and NOD2 are intracellular bacterial sensors activated by components of the bacterial peptidoglycan matrix 59. In response, these receptors assemble a signalling complex where XIAP is recruited by the receptor-proximal kinase RIPK2, leading to its ubiquitination by XIAP 60. In turn, LUBAC is recruited to the complex by the XIAP-conjugated Ub chains to enable downstream signalling 57 (Fig. 3). The formation of Met1-Ub appears to be indispensable for NOD1/NOD2-mediated inflammatory signalling and NF-κB activation, as the transcription and production of cytokines such as TNF and IL-6 is almost abolished in the absence of SHARPIN 57. Conversely, depletion of OTULIN strongly enhances the activation of NF-κB and the transcription of target genes in response to NOD2 61. Intriguingly, HOIL-1 was recently reported to be dispensable for the production of IL-6 in response to NOD2 stimulation in a murine peritonitis model 56, which might reflect distinct roles of SHARPIN and HOIL-1 for LUBAC function, at least in the context of NOD2 signalling.

### PRR – NLR family, pyrin domain-containing 3 (NLRP3)

In a recent report, HOIL-1 was shown to be needed for assembly of the NLRP3 inflammasome and secretion of IL-1β in bone marrow-derived macrophages, and *in vivo* in response to peritonisis induced by MDP (a NOD2 ligand) 56. NLRP3 senses a variety of stimuli, and its activation leads to the assembly of a multimeric inflammasome complex consisting of the adaptor protein apoptosis-associated speck-like protein containing a caspase activation and recruitment domain (ASC) and pro-caspase-1. Assembly of the inflammasome results in activation of caspase-1 and processing of the cytokines pro-IL-1β and pro-IL-18 to their bioactive forms (IL-1β and IL-18) which are secreted and contribute to proinflammatory responses 1. In response to NLRP3-activating stimuli, Met1-Ub colocalised with ASC ‘specks’, a feature of inflammasome assembly, and speck formation was dependent on HOIL-1 56. In a separate study, the JAMM domain-containing DUB BRCC36 (BRCC3 in the mouse) was found to remove Lys48-Ub and Lys63-Ub (and possibly other linkages) from NLRP3, which was required for inflammasome assembly and secretion of IL-1β 62. Met1-Ub and Lys-Ub linkages may thus have distinct regulatory functions in controlling inflammasome activity and the production of IL-1β and IL-18.

Other PRRs for which Met1-Ub has been shown to contribute to signalling include TLR2, TLR7, and TLR9, which, like IL-1βR, rely on the adaptor myeloid differentiation primary response gene 88 (MyD88) for proinflammatory signalling 15,55,63.

### PRRs – retinoic acid-inducible gene 1 (RIG-I) and melanoma differentiation-associated protein 5

In contrast to its role in NF-κB-dependent pro-inflammatory signalling, Met1-Ub appears to be a negative regulator of viral responses. Overexpression of melanoma differentiation-associated protein 5, RIG-I or mitochondrial antiviral signalling protein (MAVS) leads to activation of the interferon-stimulated response element in a manner that is repressed when LUBAC is coexpressed 64. Mouse embryonic fibroblast isolated from *cpdm* mice show increased resistance to vesicular stomatitis virus infection (decreased virus replication) and an enhanced transcriptional response to infection as compared with control cells 64. RIG-I signalling depends on the Ub ligase tripartite motif (TRIM)25 and the adaptor MAVS, and it was proposed that NEMO sequesters TRAF3 from MAVS in a LUBAC-dependent manner and attenuates the interferon-mediated antiviral response 64. However, in a separate report, LUBAC was proposed to restrict RIG-I signalling in response to Sendai virus infection by sequestering TRIM25 from RIG-I and facilitating degradative ubiquitination of TRIM25 65. Whether both models are constituents of the same mechanism through which LUBAC limits RIG-I-mediated signalling, or whether they reflect separate mechanisms, is currently not clear. Irrespective of this, it is apparent that LUBAC has a critical regulatory function in the cellular response to viral infections, and it will be important to fully elucidate the role(s) of Met1-Ub in this process.

## Met1-Ub substrates

Although protein ubiquitination in response to innate immune receptor activation is well documented 1, technical challenges initially hampered the identification of Met1-Ub linkages on substrates. The first Met1-Ub substrate to be identified was the IKK subunit NEMO 41, but recent advances in the field have led to the identification of multiple new targets.

### NEMO

NEMO is essential for activation of the IKK complex, and was first found to be a substrate for ubiquitination downstream of TNFR1, NOD2, and the adaptor protein BCL10, an essential component of the B-cell and T-cell receptor signalling complex, where, as part of the CARMA1–BCL10–MALT1 complex, BCL10 facilitates recruitment of the Ub ligase TRAF6 1,66–69. In addition to NEMO being a proposed substrate for Lys63-Ub, Met1 ubiquitination of NEMO has been reported in response to activation of TNFR, IL1βR and the CD40 receptor 14,16,24,41,43,70.

*In vitro* experiments with purified proteins have demonstrated direct ubiquitination of NEMO by LUBAC, and NEMO is ubiquitinated in cell culture when HOIL-1 and HOIP are ectopically expressed 14,21,41. NEMO interacts directly with the HOIP NZF1 domain via the region of the CoZi domain N-terminal to the Met1-Ub-binding UBAN domain, and mutation of residues involved in HOIP binding impairs its ubiquitination by LUBAC 70 (Fig. 1). These studies demonstrate that LUBAC has the capacity to conjugate Met1-Ub on NEMO *in vitro* as well as in cells.

Gerlach *et al*. (2011) combined purification of the TNFR1 signalling complex with 2D gel electrophoresis and MS to identify Met1-Ub linkages on NEMO (and RIPK1) 14. Although this conclusively showed that NEMO is a physiological substrate for Met1-Ub, the study did not investigate whether the formation of Met1-Ub on NEMO and RIPK1 was induced by the TNF treatment or whether it was a stable modification present on a small fraction of NEMO and RIPK1 molecules.

Residues within the NEMO CoZi domain, Lys285 and Lys309, have been identified as acceptor sites for LUBAC-mediated ubiquitination *in vitro*, although only Lys285 was confirmed as an acceptor site in cells 21,41. NEMO Lys285 has also been reported to be the major acceptor site for Lys63-Ub formation mediated by RIPK2 overexpression 67, indicating that Lys285 is a preferred site for NEMO ubiquitination, irrespective of the Ub chain linkage. Mutation of Lys285 and Lys309 impairs NEMO ubiquitination after IL-1β treatment and impairs NF-κB activation, suggesting that these residues, individually or in combination, are important for NEMO function 41. Interestingly, direct analysis of NEMO ubiquitination after IL-1β treatment by the use of linkage-selective DUBs *in vitro* (Ub chain restriction analysis) revealed that OTULIN did not visibly remove Ub chains formed on NEMO. In contrast, the Lys63-selective DUB AMSH-LP removed the chains, leaving only a single Ub moiety on NEMO 44. This finding challenges the view that NEMO is predominantly modified by Met1-Ub upon receptor activation, and instead suggests that little or no Met1-Ub is conjugated on NEMO after IL-1βR stimulation. The fact that NEMO binds strongly to Met1-Ub might have contributed to the notion that NEMO is a central target for Met1-Ub. It has been a common practice to evaluate NEMO ubiquitination by immunoprecipitation of NEMO followed by immunoblotting for Ub. However, under these conditions, the Met1-Ub might be copurified via the NEMO UBAN domain, and need not be covalently attached to NEMO. For example, overexpression or depletion of OTULIN strongly affects NEMO-copurified Ub chains under conditions where NEMO ubiquitination is not detectable 21,57,61. Therefore, closer inspection of NEMO as a substrate for LUBAC-mediated ubiquitination in response to innate immune receptor activation is warranted, particularly as techniques have been developed that have identified other substrates for which the Met1-Ub modification is readily detectable (see below).

### RIPK1

In response to TNFR1 stimulation, RIPK1 is a major target for ubiquitination by cIAP1 and cIAP2, which reportedly modify RIPK1 with Lys63-Ub and Lys11-Ub 71–73. In addition to these linkages, Gerlach *et al*. 14 identified Lys48-Ub and Met1-Ub on RIPK1, indicating that RIPK1 is targeted by multiple Ub ligases upon TNFR1 stimulation. *In vitro* DUB assays on TNFR1-associated ubiquitinated RIPK1 confirmed the formation of Lys63-Ub on RIPK1, whereas incubation with DUBs selective for Lys11-Ub, Lys48-Ub and Met1-Ub indicated that these linkages account for only a small fraction of the Ub chains detected 74.

Nonetheless, OTULIN overexpression strongly impairs coprecipitation of ubiquitinated RIPK1 with NEMO in TNF-treated cells, whereas small interfering RNA-mediated depletion of OTULIN increases the early accumulation of Met1-Ub on RIPK1 21. RIPK1 ubiquitination in response to TNF has also been investigated in HOIL-1-deficient fibroblasts. Whereas the overall level of ubiquitinated RIPK1 in the TNFR1 complex was similar in control and HOIL-1-deficient cells, NEMO coprecipitated less ubiquitinated RIPK1 in HOIL-1-deficient cells than in control cells 55, underlining the fact that Met1-Ub is required for proper assembly of the NEMO–RIPK1–Ub complex.

### RIPK2

RIPK2 is essential for NOD1-mediated and NOD2-mediated inflammatory responses, and multiple studies have found RIPK2 to be ubiquitinated with Lys63-linked chains 75–79. However, LUBAC activity is also required for productive NOD2 signalling 57, which prompted investigations into substrates for Met1-Ub in the context of NOD2 activation. A Met1-Ub affinity reagent based on the NEMO CoZi region was used to purify Met1-Ub-modified proteins, which were subsequently subjected to MS analysis. This approach revealed that RIPK2 is the predominant (and possibly only) protein modified by Met1-Ub in response to NOD2 stimulation 61. Accordingly, the amount of Met1-Ub on RIPK2 was decreased by overexpression of OTULIN and strongly enhanced in OTULIN-depleted cells. Furthermore, an *in vitro* DUB assay using OTULIN confirmed that ubiquinated RIPK2 contains Met1 linkages 61. Interestingly, it also revealed that only the ‘outermost’ Ub moieties were removed by OTULIN, suggesting that Met1-Ub is deposited (by LUBAC) on existing Ub chains on RIPK2 assembled through other linkages. Alternatively, Met1-Ub could be deposited on an existing mono-Ub on RIPK2 if Lys-linked Ub chains were conjugated on other Lys residues within the same RIPK2 molecules. This would yield the same processing pattern with the *in vitro* DUB assay.

### IL-1 receptor-associated kinase (IRAK1) [IL-4 receptor-associated kinase (IRAK4)/MyD88]

In a similar approach to the one used by us to identify Met1-Ub after NOD2 and TNFR1 stimulation, Cohen *et al*. used HaloTag-tagged full-length NEMO coupled to beads to capture Met1-Ub-modified proteins before and after treatment with IL-1β and Pam_3_CSK_4_ (TLR1/TLR2 agonist) 44. The study revealed that IRAK1, MyD88 and, to a lesser extent, IRAK4 are modified by Met1-Ub in response to IL-1β and Pam_3_CSK_4_. It also provided compelling evidence that Met1-Ub, at least in the context of IL-1βR stimulation, is formed on existing Lys63-Ub, and that the Lys63-Ub modification is a prerequisite for the deposition of Met1-Ub linkages by LUBAC. Conversely, impairment of LUBAC activity had little effect on the conjugation of Lys63-Ub on substrates, establishing a clear sequence of events for the deposition of individual Ub linkages within the IL-1βR complex. These observations could explain the important role of Lys63-Ub in the proinflammatory response following receptors that engage MyD88 and IL receptor-associated kinases, in contrast to it being dispensable for TNF-induced signalling. The model, however, is not in complete agreement with previous observations that the Lys63-specific E2 Ubc13 is required only for activation of MAPKs but is dispensable for IκB degradation following IL-1β treatment 50, which suggests that Met1-Ub formation is controlled independently of Lys63-Ub. Surprisingly, although NEMO is also ubiquitinated after IL-1β treatment, the Ub chains appear to predominantly consist of Lys63 linkages, with only few or no Met1 linkages 44.

Further investigation into the interplay between Met1-Ub and Lys63-Ub assembly will be important to determine how these linkages are deposited on target proteins, and whether Met1–Lys63 mixed-linkage chains have different roles from homotypic chains.

### ASC

The NLRP3 component ASC was recently reported as a LUBAC substrate 56. ASC ubiquitination was particularly evident under conditions where HOIL-1, HOIP and ASC were overexpressed, and the presence of Met1-Ub linkages on ASC was confirmed with an *in vitro* DUB assay using OTULIN. However, the amount of Met1-Ub on ASC did not appear to increase in response to NLRP3 activation, although the association of endogenous ASC with Met1-Ub was decreased in HOIL-1-deficient cells stimulated with lipopolysaccharide and nigericin (an inducer of NLRP3 inflammasome activation) 56, suggesting that LUBAC may also target other inflammasome-associated factors.

### LUBAC

Recent reports have shown that LUBAC, in particular HOIP, can also target itself for ubiquitination but that this is counterbalanced by the activity of OTULIN 21,61. Reduction of OTULIN function in cells leads to accumulation of Met1-Ub on LUBAC components, and this is further enhanced by stimulation of TNFR1 and NOD2, implying that OTULIN removes Met1-Ub from LUBAC under basal conditions and after receptor activation 22,61. In support of this, mutation of residues in the HOIP PUB domain that are essential for OTULIN binding leads to accumulation of Met1-Ub on HOIP 22.

## Future directions

In this review, we have discussed the accumulated evidence supporting a role for Met1-Ub in controlling innate immune signalling. With the discovery of LUBAC and its association with receptor signalling complexes came an appreciation that nonconventional Ub chains (i.e. other than Lys48-linked and Lys63-linked) have specialised and nonredundant functions in cell biology. Indeed, in addition to Met1-Ub in immune signalling there is emerging evidence that other linkages such as Lys11 and Lys33 linkages, regulate immune processes 73,80. In many innate immune signalling pathways, there is an inordinate number of Ub ligases relative to substrates. This is particularly evident for NOD2 signalling, where only RIPK2 could be detected in a whole-proteome approach, but where seven Ub ligases have been reported to regulate signalling (Fig. 3). This implies that each individual Ub ligase contributes to the signalling processes in a distinct manner, which might be related to the type of Ub linkage conjugated, as has been established for LUBAC. Individual Ub ligases may also favour ubiquitination of distinct Lys residues of the substrate or may be engaged after receptor activation with different kinetics. In any case, it is apparent that ubiquitination within innate immune receptor complexes is a complex process, is tightly regulated, and involves more than conjugation of conventional Ub linkages.

Much progress has been made towards understanding the function and regulation of Met1-Ub, but there are still several outstanding questions that we consider to be particularly important. LUBAC seems to be constantly active in cells, but the accumulation of Met1-Ub is continuously counterbalanced by OTULIN bound to HOIP. However, upon receptor activation, Met1-Ub accumulates on specific target proteins. This suggests there is a regulated mechanism to ‘release’ LUBAC from OTULIN, to direct its activity selectively to certain substrates, and subsequently to protect the formed Met1-Ub chains from OTULIN. This is especially obvious in the context of NOD2 stimulation, where Met1-Ub accumulates solely on RIPK2 under conditions where OTULIN is present, and is strongly increased in amount and spreads to additional proteins when OTULIN activity is diminished 22,61. Recent data showing phosphorylation-regulated interaction of OTULIN and LUBAC supports the notion of a ‘release’ mechanism, but this most likely does not explain why certain Met1-Ub chains appear to be protected from OTULIN, whereas others are readily disassembled. A better understanding of the underlying mechanisms governing the deposition and accumulation of Met1-Ub (as well as other linkages), in particular the identification and characterisation of the kinase(s)/phosphatase(s) controlling the OTULIN–LUBAC interaction, will provide important insights into how innate immune signalling is regulated. In this regard, other proteins that interact with the PUB domain in HOIP, such as the AAA+ ATPase valosin-containing protein (also termed p97) 22,23 and CYLD 24, may also affect LUBAC function and Met1-Ub formation.

The generation of mixed-linkage Ub chains such as Met1/Lys63-Ub chains is an emerging and highly interesting concept. Most Ub-binding domains recognise 1–2 Ub moieties, and even in proteins where the domains exist in tandem or when they are brought together through protein dimerization, Ub chains of approximately four Ub units are sufficient for efficient binding 40,81,82. This has led to speculations about how long Ub chains have to be to support productive signalling, particularly because stimulation of innate immune receptors often leads to a dramatic increase in the apparent molecular mass of the ubiquitinated substrate(s). Mixed-linkage Ub chains could provide an elegant solution to this conundrum, as these chains would be able to accommodate the binding of several linkage-selective signalling complexes, such as TGF-β-activated kinase 1 (TAK1)-binding protein (TAB)–TAK1 and NEMO–IKK complexes. Another intriguing possibility is that mixed Ub chains constitute unique signals in the cell, and that there are specialised ‘readers’ with the capacity to distinguish between mixed-linkage and homotypic Ub chains.

The current evidence indicates that Met1-Ub has diverse roles and/or levels of importance in NF-κB and MAPK signalling, depending on the innate immune receptor. This is somewhat surprising, as NEMO and its Ub-binding capacity appear to be crucial for signalling, irrespective of the receptor system engaged 29,83,84. Could this be explained by redundancy and LUBAC not being the only Ub ligase that assembles Met1-Ub? Or can extensive formation of Lys63-Ub (or another Ub linkage) in certain receptor complexes override the requirement of Met1-Ub for IKK activation?

The emerging notion that Met1-Ub serves cellular functions beyond innate immune signalling is underscored by the early embryonic lethality and the observed developmental defects of mice with a deficiency in LUBAC activity 44 and mice harbouring inactivating mutations in OTULIN 7 (Table 1). Thus, we have undoubtedly only just started to understand the complex nature of Met1-linked Ub chains and how they are regulated.

## Abbreviations

ABIN: A20 binding and inhibitor of nuclear factor-κB
ASC: apoptosis-associated speck-like protein containing a caspase activation and recruitment domain
BCR: B-cell receptor
CBR: catalytic in-between-RING
CC: coiled coil
CoZi: coiled-coil and zinc finger
CYLD: cylindromatosis tumour suppressor
DLBCL: diffuse large B-cell lymphoma
DUB: deubiquitinase
HOIL-1: haem-oxidized IRP2 ubiquitin ligase-1
HOIP: haem-oxidized IRP2 ubiquitin ligase-1-interacting protein
IAP: inhibitor of apoptosis
IBR: in-between-RING
IKK: IκB kinase complex
IL: interleukin
IL-1βR: interkeukin-1β receptor
IRAK1: interleukin-1 receptor-associated kinase
IRAK4: interleukin-4 receptor-associated kinase
LDD: linear ubiquitin chain-determining domain
LUBAC: linear ubiquitin chain assembly complex
MAPK: mitogen-activated protein kinase
MAVS: mitochondrial antiviral signaling protein
Met1-Ub: N-terminal methionine-linked ubiquitin
MyD88: myeloid differentiation primary response gene 88
NEMO: nuclear factor-κB essential modifier
NF-Bκ: nuclear factor-κB
NLRP3: NLR family, pyrin domain-containing 3
NOD: nucleotide-binding and oligomerisation domain-containing protein
NZF: Npl4 zinc finger
OTU: ovarian tumour
PIM: peptide:*N*-glycanase/ubiquitin-associated UBA-containing or UBX-containing protein-interacting motif
PRR: pattern recognition receptor
PUB: peptide:*N*-glycanase/ubiquitin-associated UBA-containing or UBX-containing protein
RIG-I: retinoic acid-inducible gene 1
RIPK: receptor-interacting protein kinase
SHARPIN: SHANK-associated RH domain interactor
SNP: single-nucleotide polymorphism
TAB: transforming growth factor-β-activated kinase 1-binding protein
TAK1: transforming growth factor-β-activated kinase 1
TLR: Toll-like receptor
TNF: tumour necrosis factor
TNFR1: tumour necrosis factor receptor 1
TRAF: tumour necrosis factor receptor-associated factor
TRIM: tripartite motif
Ub: ubiquitin
UBA: ubiquitin-associated
UBAN: ubiquitin binding in A20 binding and inhibitor of nuclear factor-κB and nuclear factor-κB essential modifier
UBL: ubiquitin-like

## References

[1] Jiang X, Chen ZJ (2012). The role of ubiquitylation in immune defence and pathogen evasion. Nat Rev Immunol.

[2] Newton K, Dixit VM (2012). Signaling in innate immunity and inflammation. Cold Spring Harbor Perspect Biol.

[3] Takeuchi O, Akira S (2010). Pattern recognition receptors and inflammation. Cell.

[4] Damgaard RB, Gyrd-Hansen M (2011). Inhibitor of apoptosis (IAP) proteins in regulation of inflammation and innate immunity. Discov Med.

[5] Baud V, Karin M (2009). Is NF-kappaB a good target for cancer therapy? Hopes and pitfalls. Nat Rev Drug Discov.

[6] Kulathu Y, Komander D (2012). Atypical ubiquitylation – the unexplored world of polyubiquitin beyond Lys48 and Lys63 linkages. Nat Rev Mol Cell Biol.

[7] Rivkin E, Almeida SM, Ceccarelli DF, Juang YC, MacLean TA, Srikumar T, Huang H, Dunham WH, Fukumura R, Xie G (2013). The linear ubiquitin-specific deubiquitinase gumby regulates angiogenesis. Nature.

[8] Mackay C, Carroll E, Ibrahim AF, Garg A, Inman GJ, Hay RT, Alpi AF (2014). E3 ubiquitin ligase HOIP attenuates apoptotic cell death induced by cisplatin. Cancer Res.

[9] Niu J, Shi Y, Iwai K, Wu ZH (2011). LUBAC regulates NF-kappaB activation upon genotoxic stress by promoting linear ubiquitination of NEMO. EMBO J.

[10] Ozkaynak E, Finley D, Varshavsky A (1984). The yeast ubiquitin gene: head-to-tail repeats encoding a polyubiquitin precursor protein. Nature.

[11] Baker RT, Board PG (1987). The human ubiquitin gene family: structure of a gene and pseudogenes from the Ub B subfamily. Nucleic Acids Res.

[12] Wiborg O, Pedersen MS, Wind A, Berglund LE, Marcker KA, Vuust J (1985). The human ubiquitin multigene family: some genes contain multiple directly repeated ubiquitin coding sequences. EMBO J.

[13] Amerik A, Swaminathan S, Krantz BA, Wilkinson KD, Hochstrasser M (1997). In vivo disassembly of free polyubiquitin chains by yeast Ubp14 modulates rates of protein degradation by the proteasome. EMBO J.

[14] Gerlach B, Cordier SM, Schmukle AC, Emmerich CH, Rieser E, Haas TL, Webb AI, Rickard JA, Anderton H, Wong WW (2011). Linear ubiquitination prevents inflammation and regulates immune signalling. Nature.

[15] Ikeda F, Deribe YL, Skanland SS, Stieglitz B, Grabbe C, Franz-Wachtel M, van Wijk SJ, Goswami P, Nagy V, Terzic J (2011). SHARPIN forms a linear ubiquitin ligase complex regulating NF-kappaB activity and apoptosis. Nature.

[16] Tokunaga F, Nakagawa T, Nakahara M, Saeki Y, Taniguchi M, Sakata S, Tanaka K, Nakano H, Iwai K (2011). SHARPIN is a component of the NF-kappaB-activating linear ubiquitin chain assembly complex. Nature.

[17] Stieglitz B, Morris-Davies AC, Koliopoulos MG, Christodoulou E, Rittinger K (2012). LUBAC synthesizes linear ubiquitin chains via a thioester intermediate. EMBO Rep.

[18] Kirisako T, Kamei K, Murata S, Kato M, Fukumoto H, Kanie M, Sano S, Tokunaga F, Tanaka K, Iwai K (2006). A ubiquitin ligase complex assembles linear polyubiquitin chains. EMBO J.

[19] Stieglitz B, Rana RR, Koliopoulos MG, Morris-Davies AC, Schaeffer V, Christodoulou E, Howell S, Brown NR, Dikic I, Rittinger K (2013). Structural basis for ligase-specific conjugation of linear ubiquitin chains by HOIP. Nature.

[20] Smit JJ, Monteferrario D, Noordermeer SM, van Dijk WJ, van der Reijden BA, Sixma TK (2012). The E3 ligase HOIP specifies linear ubiquitin chain assembly through its RING-IBR-RING domain and the unique LDD extension. EMBO J.

[21] Keusekotten K, Elliott PR, Glockner L, Fiil BK, Damgaard RB, Kulathu Y, Wauer T, Hospenthal MK, Gyrd-Hansen M, Krappmann D (2013). OTULIN antagonizes LUBAC signaling by specifically hydrolyzing Met1-linked polyubiquitin. Cell.

[22] Elliott PR, Nielsen SV, Marco-Casanova P, Fiil BK, Keusekotten K, Mailand N, Freund SM, Gyrd-Hansen M, Komander D (2014). Molecular basis and regulation of OTULIN–LUBAC interaction. Mol Cell.

[23] Schaeffer V, Akutsu M, Olma MH, Gomes LC, Kawasaki M, Dikic I (2014). Binding of OTULIN to the PUB domain of HOIP controls NF-kappaB signaling. Mol Cell.

[24] Takiuchi T, Nakagawa T, Tamiya H, Fujita H, Sasaki Y, Saeki Y, Takeda H, Sawasaki T, Buchberger A, Kimura T (2014). Suppression of LUBAC-mediated linear ubiquitination by a specific interaction between LUBAC and the deubiquitinases CYLD and OTULIN. Genes Cells.

[25] Bremm A, Freund SM, Komander D (2010). Lys11-linked ubiquitin chains adopt compact conformations and are preferentially hydrolyzed by the deubiquitinase Cezanne. Nat Struct Mol Biol.

[26] Komander D, Reyes-Turcu F, Licchesi JD, Odenwaelder P, Wilkinson KD, Barford D (2009). Molecular discrimination of structurally equivalent Lys 63-linked and linear polyubiquitin chains. EMBO Rep.

[27] Eddins MJ, Varadan R, Fushman D, Pickart CM, Wolberger C (2007). Crystal structure and solution NMR studies of Lys48-linked tetraubiquitin at neutral pH. J Mol Biol.

[28] Varadan R, Assfalg M, Haririnia A, Raasi S, Pickart C, Fushman D (2004). Solution conformation of Lys63-linked di-ubiquitin chain provides clues to functional diversity of polyubiquitin signaling. J Biol Chem.

[29] Rahighi S, Ikeda F, Kawasaki M, Akutsu M, Suzuki N, Kato R, Kensche T, Uejima T, Bloor S, Komander D (2009). Specific recognition of linear ubiquitin chains by NEMO is important for NF-kappaB activation. Cell.

[30] Laplantine E, Fontan E, Chiaravalli J, Lopez T, Lakisic G, Veron M, Agou F, Israel A (2009). NEMO specifically recognizes K63-linked poly-ubiquitin chains through a new bipartite ubiquitin-binding domain. EMBO J.

[31] Lo YC, Lin SC, Rospigliosi CC, Conze DB, Wu CJ, Ashwell JD, Eliezer D, Wu H (2009). Structural basis for recognition of diubiquitins by NEMO. Mol Cell.

[32] Kensche T, Tokunaga F, Ikeda F, Goto E, Iwai K, Dikic I (2012). Analysis of nuclear factor-kappaB (NF-kappaB) essential modulator (NEMO) binding to linear and lysine-linked ubiquitin chains and its role in the activation of NF-kappaB. J Biol Chem.

[33] Filipe-Santos O, Bustamante J, Haverkamp MH, Vinolo E, Ku CL, Puel A, Frucht DM, Christel K, von Bernuth H, Jouanguy E (2006). X-linked susceptibility to mycobacteria is caused by mutations in NEMO impairing CD40-dependent IL-12 production. J Exp Med.

[34] Doffinger R, Smahi A, Bessia C, Geissmann F, Feinberg J, Durandy A, Bodemer C, Kenwrick S, Dupuis-Girod S, Blanche S (2001). X-linked anhidrotic ectodermal dysplasia with immunodeficiency is caused by impaired NF-kappaB signaling. Nat Genet.

[35] Bloor S, Ryzhakov G, Wagner S, Butler PJ, Smith DL, Krumbach R, Dikic I, Randow F (2008). Signal processing by its coil zipper domain activates IKK gamma. Proc Natl Acad Sci USA.

[36] Nanda SK, Venigalla RK, Ordureau A, Patterson-Kane JC, Powell DW, Toth R, Arthur JS, Cohen P (2011). Polyubiquitin binding to ABIN1 is required to prevent autoimmunity. J Exp Med.

[37] Wagner S, Carpentier I, Rogov V, Kreike M, Ikeda F, Lohr F, Wu CJ, Ashwell JD, Dotsch V, Dikic I (2008). Ubiquitin binding mediates the NF-kappaB inhibitory potential of ABIN proteins. Oncogene.

[38] Oshima S, Turer EE, Callahan JA, Chai S, Advincula R, Barrera J, Shifrin N, Lee B, Benedict Yen TS, Woo T (2009). ABIN-1 is a ubiquitin sensor that restricts cell death and sustains embryonic development. Nature.

[39] Haas TL, Emmerich CH, Gerlach B, Schmukle AC, Cordier SM, Rieser E, Feltham R, Vince J, Warnken U, Wenger T (2009). Recruitment of the linear ubiquitin chain assembly complex stabilizes the TNF-R1 signaling complex and is required for TNF-mediated gene induction. Mol Cell.

[40] Gyrd-Hansen M, Darding M, Miasari M, Santoro MM, Zender L, Xue W, Tenev T, da Fonseca PC, Zvelebil M, Bujnicki JM (2008). IAPs contain an evolutionarily conserved ubiquitin-binding domain that regulates NF-kappaB as well as cell survival and oncogenesis. Nat Cell Biol.

[41] Tokunaga F, Sakata S, Saeki Y, Satomi Y, Kirisako T, Kamei K, Nakagawa T, Kato M, Murata S, Yamaoka S (2009). Involvement of linear polyubiquitylation of NEMO in NF-kappaB activation. Nat Cell Biol.

[42] Seymour RE, Hasham MG, Cox GA, Shultz LD, Hogenesch H, Roopenian DC, Sundberg JP (2007). Spontaneous mutations in the mouse Sharpin gene result in multiorgan inflammation, immune system dysregulation and dermatitis. Genes Immun.

[43] Sasaki Y, Sano S, Nakahara M, Murata S, Kometani K, Aiba Y, Sakamoto S, Watanabe Y, Tanaka K, Kurosaki T (2013). Defective immune responses in mice lacking LUBAC-mediated linear ubiquitination in B cells. EMBO J.

[44] Emmerich CH, Ordureau A, Strickson S, Arthur JS, Pedrioli PG, Komander D, Cohen P (2013). Activation of the canonical IKK complex by K63/M1-linked hybrid ubiquitin chains. Proc Natl Acad Sci USA.

[45] Yang Y, Schmitz R, Mitala J, Whiting A, Xiao W, Ceribelli M, Wright GW, Zhao H, Yang Y, Xu W (2014). Essential role of the linear ubiquitin chain assembly complex in lymphoma revealed by rare germline polymorphisms. Cancer Discov.

[46] Compagno M, Lim WK, Grunn A, Nandula SV, Brahmachary M, Shen Q, Bertoni F, Ponzoni M, Scandurra M, Califano A (2009). Mutations of multiple genes cause deregulation of NF-kappaB in diffuse large B-cell lymphoma. Nature.

[47] Davis RE, Ngo VN, Lenz G, Tolar P, Young RM, Romesser PB, Kohlhammer H, Lamy L, Zhao H, Yang Y (2010). Chronic active B-cell-receptor signalling in diffuse large B-cell lymphoma. Nature.

[48] Xu M, Skaug B, Zeng W, Chen ZJ (2009). A ubiquitin replacement strategy in human cells reveals distinct mechanisms of IKK activation by TNFalpha and IL-1beta. Mol Cell.

[49] Lomaga MA, Yeh WC, Sarosi I, Duncan GS, Furlonger C, Ho A, Morony S, Capparelli C, Van G, Kaufman S (1999). TRAF6 deficiency results in osteopetrosis and defective interleukin-1, CD40, and LPS signaling. Genes Dev.

[50] Yamamoto M, Okamoto T, Takeda K, Sato S, Sanjo H, Uematsu S, Saitoh T, Yamamoto N, Sakurai H, Ishii KJ (2006). Key function for the Ubc13 E2 ubiquitin-conjugating enzyme in immune receptor signaling. Nat Immunol.

[51] Deng L, Wang C, Spencer E, Yang L, Braun A, You J, Slaughter C, Pickart C, Chen ZJ (2000). Activation of the IkappaB kinase complex by TRAF6 requires a dimeric ubiquitin-conjugating enzyme complex and a unique polyubiquitin chain. Cell.

[52] Ordureau A, Smith H, Windheim M, Peggie M, Carrick E, Morrice N, Cohen P (2008). The IRAK-catalysed activation of the E3 ligase function of Pellino isoforms induces the Lys63-linked polyubiquitination of IRAK1. Biochem J.

[53] Kim TW, Yu M, Zhou H, Cui W, Wang J, DiCorleto P, Fox P, Xiao H, Li X (2012). Pellino 2 is critical for Toll-like receptor/interleukin-1 receptor (TLR/IL-1R)-mediated post-transcriptional control. J Biol Chem.

[54] Tarantino N, Tinevez JY, Crowell EF, Boisson B, Henriques R, Mhlanga M, Agou F, Israel A, Laplantine E (2014). TNF and IL-1 exhibit distinct ubiquitin requirements for inducing NEMO–IKK supramolecular structures. J Cell Biol.

[55] Boisson B, Laplantine E, Prando C, Giliani S, Israelsson E, Xu Z, Abhyankar A, Israel L, Trevejo-Nunez G, Bogunovic D (2012). Immunodeficiency, autoinflammation and amylopectinosis in humans with inherited HOIL-1 and LUBAC deficiency. Nat Immunol.

[56] Rodgers MA, Bowman JW, Fujita H, Orazio N, Shi M, Liang Q, Amatya R, Kelly TJ, Iwai K, Ting J (2014). The linear ubiquitin assembly complex (LUBAC) is essential for NLRP3 inflammasome activation. J Exp Med.

[57] Damgaard RB, Nachbur U, Yabal M, Wong WW, Fiil BK, Kastirr M, Rieser E, Rickard JA, Bankovacki A, Peschel C (2012). The ubiquitin ligase XIAP recruits LUBAC for NOD2 signaling in inflammation and innate immunity. Mol Cell.

[58] Warner N, Burberry A, Franchi L, Kim YG, McDonald C, Sartor MA, Nunez G (2013). A genome-wide siRNA screen reveals positive and negative regulators of the NOD2 and NF-kappaB signaling pathways. Sci Signal.

[59] Chen G, Shaw MH, Kim YG, Nunez G (2009). NOD-like receptors: role in innate immunity and inflammatory disease. Annu Rev Pathol.

[60] Damgaard RB, Fiil BK, Speckmann C, Yabal M, zur Stadt U, Bekker-Jensen S, Jost PJ, Ehl S, Mailand N, Gyrd-Hansen M (2013). Disease-causing mutations in the XIAP BIR2 domain impair NOD2-dependent immune signalling. EMBO Mol Med.

[61] Fiil BK, Damgaard RB, Wagner SA, Keusekotten K, Fritsch M, Bekker-Jensen S, Mailand N, Choudhary C, Komander D, Gyrd-Hansen M (2013). OTULIN restricts Met1-linked ubiquitination to control innate immune signaling. Mol Cell.

[62] Py BF, Kim MS, Vakifahmetoglu-Norberg H, Yuan J (2013). Deubiquitination of NLRP3 by BRCC3 critically regulates inflammasome activity. Mol Cell.

[63] Zak DE, Schmitz F, Gold ES, Diercks AH, Peschon JJ, Valvo JS, Niemisto A, Podolsky I, Fallen SG, Suen R (2011). Systems analysis identifies an essential role for SHANK-associated RH domain-interacting protein (SHARPIN) in macrophage Toll-like receptor 2 (TLR2) responses. Proc Natl Acad Sci USA.

[64] Belgnaoui SM, Paz S, Samuel S, Goulet ML, Sun Q, Kikkert M, Iwai K, Dikic I, Hiscott J, Lin R (2012). Linear ubiquitination of NEMO negatively regulates the interferon antiviral response through disruption of the MAVS–TRAF3 complex. Cell Host Microbe.

[65] Inn KS, Gack MU, Tokunaga F, Shi M, Wong LY, Iwai K, Jung JU (2011). Linear ubiquitin assembly complex negatively regulates RIG-I- and TRIM25-mediated type I interferon induction. Mol Cell.

[66] Tang ED, Wang CY, Xiong Y, Guan KL (2003). A role for NF-kappaB essential modifier/IkappaB kinase-gamma (NEMO/IKKgamma) ubiquitination in the activation of the IkappaB kinase complex by tumor necrosis factor-alpha. J Biol Chem.

[67] Abbott DW, Wilkins A, Asara JM, Cantley LC (2004). The Crohn's disease protein, NOD2, requires RIP2 in order to induce ubiquitinylation of a novel site on NEMO. Curr Biol.

[68] Sun L, Deng L, Ea CK, Xia ZP, Chen ZJ (2004). The TRAF6 ubiquitin ligase and TAK1 kinase mediate IKK activation by BCL10 and MALT1 in T lymphocytes. Mol Cell.

[69] Zhou H, Wertz I, O'Rourke K, Ultsch M, Seshagiri S, Eby M, Xiao W, Dixit VM (2004). Bcl10 activates the NF-kappaB pathway through ubiquitination of NEMO. Nature.

[70] Fujita H, Rahighi S, Akita M, Kato R, Sasaki Y, Wakatsuki S, Iwai K (2014). Mechanism underlying IkappaB kinase activation mediated by the linear ubiquitin chain assembly complex. Mol Cell Biol.

[71] Bertrand MJ, Milutinovic S, Dickson KM, Ho WC, Boudreault A, Durkin J, Gillard JW, Jaquith JB, Morris SJ, Barker PA (2008). cIAP1 and cIAP2 facilitate cancer cell survival by functioning as E3 ligases that promote RIP1 ubiquitination. Mol Cell.

[72] Varfolomeev E, Goncharov T, Fedorova AV, Dynek JN, Zobel K, Deshayes K, Fairbrother WJ, Vucic D (2008). c-IAP1 and c-IAP2 are critical mediators of tumor necrosis factor alpha (TNFalpha)-induced NF-kappaB activation. J Biol Chem.

[73] Dynek JN, Goncharov T, Dueber EC, Fedorova AV, Izrael-Tomasevic A, Phu L, Helgason E, Fairbrother WJ, Deshayes K, Kirkpatrick DS (2010). c-IAP1 and UbcH5 promote K11-linked polyubiquitination of RIP1 in TNF signalling. EMBO J.

[74] Mevissen TE, Hospenthal MK, Geurink PP, Elliott PR, Akutsu M, Arnaudo N, Ekkebus R, Kulathu Y, Wauer T, El Oualid F (2013). OTU deubiquitinases reveal mechanisms of linkage specificity and enable ubiquitin chain restriction analysis. Cell.

[75] Abbott DW, Yang Y, Hutti JE, Madhavarapu S, Kelliher MA, Cantley LC (2007). Coordinated regulation of Toll-like receptor and NOD2 signaling by K63-linked polyubiquitin chains. Mol Cell Biol.

[76] Hasegawa M, Fujimoto Y, Lucas PC, Nakano H, Fukase K, Nunez G, Inohara N (2008). A critical role of RICK/RIP2 polyubiquitination in Nod-induced NF-kappaB activation. EMBO J.

[77] Yang Y, Yin C, Pandey A, Abbott D, Sassetti C, Kelliher MA (2007). NOD2 pathway activation by MDP or Mycobacterium tuberculosis infection involves the stable polyubiquitination of Rip2. J Biol Chem.

[78] Tao M, Scacheri PC, Marinis JM, Harhaj EW, Matesic LE, Abbott DW (2009). ITCH K63-ubiquitinates the NOD2 binding protein, RIP2, to influence inflammatory signaling pathways. Curr Biol.

[79] Yang S, Wang B, Humphries F, Jackson R, Healy ME, Bergin R, Aviello G, Hall B, McNamara D, Darby T (2013). Pellino3 ubiquitinates RIP2 and mediates Nod2-induced signaling and protective effects in colitis. Nat Immunol.

[80] Huang H, Jeon MS, Liao L, Yang C, Elly C, Yates JR, Liu YC (2010). K33-linked polyubiquitination of T cell receptor-zeta regulates proteolysis-independent T cell signaling. Immunity.

[81] Dikic I, Wakatsuki S, Walters KJ (2009). Ubiquitin-binding domains – from structures to functions. Nat Rev Mol Cell Biol.

[82] Raasi S, Varadan R, Fushman D, Pickart CM (2005). Diverse polyubiquitin interaction properties of ubiquitin-associated domains. Nat Struct Mol Biol.

[83] Rudolph D, Yeh WC, Wakeham A, Rudolph B, Nallainathan D, Potter J, Elia AJ, Mak TW (2000). Severe liver degeneration and lack of NF-kappaB activation in NEMO/IKKgamma-deficient mice. Genes Dev.

[84] Schmidt-Supprian M, Bloch W, Courtois G, Addicks K, Israel A, Rajewsky K, Pasparakis M (2000). NEMO/IKK gamma-deficient mice model incontinentia pigmenti. Mol Cell.

